# Chicken infectious anemia virus exploits host CK2α as an essential factor for its replication via VP2 Ser182/Asp183-mediated interaction

**DOI:** 10.1128/jvi.01739-25

**Published:** 2026-03-31

**Authors:** Ziyue Ma, Hongnuan Wang, Yongqiang Wang, Xiaoqi Li, Hong Cao, Li Gao, Shijun J. Zheng

**Affiliations:** 1 National Key Laboratory of Veterinary Public Health Security, China Agricultural University 34752, Beijing, China; 2 Key Laboratory of Animal Epidemiology of the Ministry of Agriculture, China Agricultural University 34752, Beijing, China; 3 College of Veterinary Medicine, China Agricultural University 34752, Beijing, China; College of Agriculture & Life Sciences, University of Arizona, Tucson, Arizona, USA

**Keywords:** CIAV, VP2, CK2α, viral replication

## Abstract

Chicken infectious anemia virus (CIAV) induces severe immunosuppression in chickens. The nonstructural protein VP2 of CIAV is shown to play critical roles in viral assembly and viral replication. However, the molecular mechanisms by which CIAV VP2 hijacks host machinery to promote efficient viral replication remain elusive. Here, we showed that casein kinase 2 alpha (CK2α) subunit was identified to interact with CIAV VP2 to promote viral replication. We found that knockdown of cellular CK2α by RNAi in MDCC-MSB1 or inhibition of its kinase activity by an inhibitor significantly suppressed CIAV replication in cells. Furthermore, we verified that CK2α stabilized VP2 by inhibiting its proteasomal degradation. In addition, we demonstrated that Ser182 and Asp183 of VP2 served as essential residues for CK2α binding. Using reverse genetics technique, we rescued the recombinant virus with mutations at Ser182 and Asp183 of VP2 and demonstrated that mutations at these sites disrupted VP2-CK2α binding, reduced viral replication, and mitigated CIAV-induced bone marrow hypoplasia and thymic damage *in vivo*. Our results indicate that CIAV exploits cellular CK2α for its replication and pathogenesis, implying that CK2α can be used as a potential target for the development of antiviral drugs in effective control of CIAV infection.

**IMPORTANCE:**

Chicken infectious anemia virus (CIAV) causes severe immunosuppression and substantial economic losses in the global poultry industry. Current strategies to control CIAV infection are still limited. This study identifies host kinase casein kinase 2 alpha (CK2α) as a key cellular factor that binds and stabilizes CIAV VP2, promoting viral replication. Inhibition or knockdown of CK2α suppressed viral replication. The rescued virus with VP2 S182A/D183A mutations exhibited reduced viral replication and attenuated pathogenicity. These findings reveal a crucial mechanism whereby CIAV VP2 exploits host CK2α for efficient viral replication, highlighting the VP2-CK2α interaction as a potential target for developing novel antiviral strategies.

## INTRODUCTION

Chicken infectious anemia virus (CIAV), a member of the *Gyrovirus* genus in the *Anelloviridae* family (1), is an immunosuppressive pathogen that induces thymic atrophy and bone marrow hypoplasia in infected chickens (2). Since its first identification in 1979 (3), CIAV has been found worldwide and is responsible for substantial economic losses (4–7). However, studies on CIAV have mainly focused on its isolation and identification (8–10), co-infection with other diseases (11–13), or vaccine development (14–16). The molecular mechanisms by which CIAV exploits host cellular factors to enable its replication are currently poorly understood.

CIAV is a nonenveloped virus with a single-stranded DNA genome encoding one structural protein (VP1) and two nonstructural proteins (VP2 and VP3). The virus replicates in lymphoid cells and is cytopathic (17). Cell death is attributed to apoptosis induced by VP3 expression (18, 19). Lymphocyte depletion results in immunosuppression and increased susceptibility to various viral and bacterial pathogens (20). The nonstructural protein VP2 serves as a multifunctional scaffold protein during viral assembly, which is essential for VP1 folding and the proper exposure of its neutralizing epitopes (21, 22). Additionally, VP2 exhibits phosphatase activity, which regulates virus-related cellular processes (23). Recent studies have indicated that VP2 may also play a role in modulating viral replication and the host antiviral response. For example, mutagenesis studies targeting the dual-specificity phosphatase (DSP) catalytic motif of VP2, such as the K102D mutation, have been shown to significantly repress viral replication, underscoring the critical role of VP2 phosphatase activity in promoting viral growth (21). Despite these advances, the molecular mechanisms by which CIAV VP2 hijacks host factors to promote efficient viral replication remain elusive.

Casein kinase 2 (CK2) is a constitutively active serine/threonine kinase expressed in eukaryotic cells (24). It typically assembles as a heterotetrameric holoenzyme composed of two catalytic subunits (α/α') and two regulatory β subunits (25). Proteomic analyses indicate that CK2α is the predominantly expressed catalytic subunit (26, 27). CK2 exhibits broad functional versatility in viral pathogenesis. For example, CK2 phosphorylates human papillomavirus 16 E2 to promote its interaction with TopBP1, thereby stabilizing E2 and facilitating the viral life cycle (28). Similarly, CK2α regulates hepatitis B virus core protein stability and promotes viral replication via phosphorylation of NSP5 (29, 30). During SARS-CoV-2 infection, CK2 activity is enhanced, and pharmacological CK2 inhibitors have been shown to have potent antiviral efficacy (31). Recent evidence further identifies CK2 as a critical host kinase regulating influenza virus nuclear export protein function (32), establishing its role as a central regulator of viral adaptation. Its conserved kinase activity makes CK2 a common host factor exploited by diverse viruses to manipulate host pathways and promote their infection.

In this study, we identified that CK2α was a novel host factor that interacts with VP2, and knockdown of CK2α or inhibition of CK2α kinase activity inhibited CIAV replication. Interestingly, our data showed that VP2-CK2α interplay blocked VP2 degradation by the proteasome, and that Ser182 and Asp183 of VP2 were essential for CK2α binding and stabilization. Importantly, the rescued CIAV with VP2 mutation at sites Ser182 and Asp183 displayed impaired replication *in vitro* and attenuated pathogenesis *in vivo*. These findings imply that CK2α plays a critical role in CIAV replication and pathogenesis.

## RESULTS

### CIAV VP2 interacts with the cellular protein CK2α

**Fig 1:**
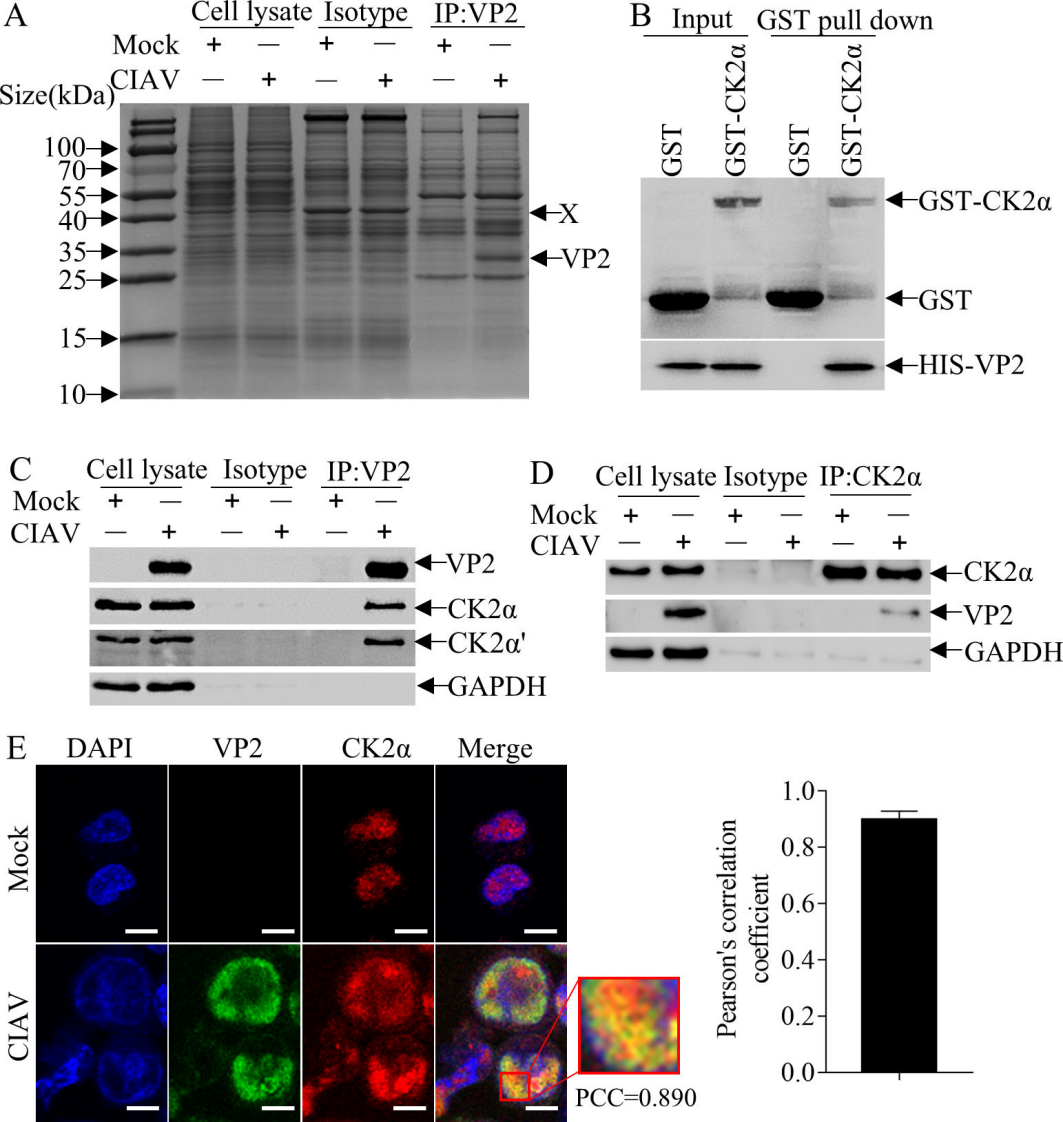
CIAV VP2 interacts with the cellular protein CK2α. (**A**) Pull-down analysis of VP2-interacting proteins in CIAV-infected MDCC-MSB1 cells. MDCC-MSB1 cells were either mock-infected or infected with CIAV. Cell lysates were subjected to pull-down using an anti-VP2 monoclonal antibody, and proteins were separated by SDS-PAGE. The protein band indicated by the arrow was excised and analyzed by LC-MS/MS to identify VP2-interacting partners. (**B**) Direct interaction between VP2 and CK2α. The recombinant glutathione S-transferase (GST)-CK2α protein was bound to anti-GST agarose beads at 4°C for 2 h, with the GST protein used as a control. The beads were incubated with His-tagged VP2 overnight at 4°C, after which the samples were eluted and analyzed by Western blot with anti-His and anti-GST antibodies. (**C and D**) Validation of the interaction between VP2 and endogenous CK2α in CIAV-infected cells. MDCC-MSB1 cells were mock-infected or infected with CIAV at a multiplicity of infection (MOI) of 1. At 48 h post-infection, co-immunoprecipitation (co-IP) assays were performed using (**C**) an anti-VP2 monoclonal antibody or an (**D**) anti-CK2α monoclonal antibody. Immune complexes were analyzed by Western blot: (**C**) CK2α was detected using an anti-CK2α antibody, CK2α′ was detected using an anti-CK2α′ antibody; (**D**) VP2 was detected using an anti-VP2 antibody. “Isotype” denotes the IgG1 isotype control antibody for the respective primary antibody (anti-VP2 or anti-CK2α). (**E**) Colocalization of CIAV VP2 and endogenous CK2α in CIAV-infected cells. MDCC-MSB1 cells were mock-infected or infected with CIAV (MOI = 1). At 24 h post-infection, cells were fixed, permeabilized, and stained with a mouse anti-VP2 antibody and a rabbit anti-CK2α antibody. Secondary antibodies used were fluorescein isothiocyanate (FITC)-conjugated goat anti-mouse IgG (green, for VP2) and tetramethylrhodamine isothiocyanate (TRITC)-conjugated goat anti-rabbit IgG (red, for CK2α). Nuclei were counterstained with 4',6-diamidino-2-phenylindole (DAPI, blue). Images were acquired using a confocal laser scanning microscope. Scale bar = 10 µm. Colocalization was assessed by calculating the Pearson correlation coefficient with ImageJ/Fiji software.

To explore the mechanisms by which VP2 contributes to CIAV infection, we infected MDCC-MSB1 (MSB1) cells with CIAV and performed a pull-down assay using anti-VP2 monoclonal antibody (mAb). As shown in Fig. 1A, proteins of approximately 40 kDa were significantly enriched in the presence of VP2, suggesting that proteins of this molecular weight may interact with VP2. Liquid chromatography‐tandem mass spectrometry (LC-MS/MS) was further conducted to identify specific proteins. A total of 43 candidate proteins were identified in the infected group (Tables S1 and S2). Among these proteins, CK2α was identified with a high score based on multiple identification metrics, including the abundance of unique peptides, sequence coverage, and spectral quality. Its sequence coverage was 30.4% (Table 1). Thus, CK2α was selected for further investigation.

**TABLE 1:** Identification of proteins by mass spectrometry analysis

Protein	Score	Coverage (%)	Unique peptides
Casein kinase 2 subunit alpha	273.35	30.4	12
Histone H1.11R, histone H1.01, H15 domain-containing protein	33.684	10	2
Elongation factor Tu, mitochondrial, elongation factor Tu	28.427	11.9	4
OVA (Fragment)	25.404	8.5	2
Myosin-9	22.569	1.2	2
L-lactate dehydrogenase, L-lactate dehydrogenase A chain	21.411	4.9	1
S-phase kinase-associated protein 2, F-box domain-containing protein	20.813	7.5	3
Actin-related protein 2	17.592	3.2	1
Tubulin domain-containing protein, Tubulin alpha chain	17.544	7.8	1
Actin, alpha skeletal muscle, actin, gamma-enteric smooth muscle	11.541	25.7	1

To verify a direct interaction, we performed an *in vitro* GST pull-down assay using purified proteins. GST-CK2α, but not GST alone, specifically pulled down His-tagged VP2 (Fig. 1B), confirming their direct binding in the absence of other cellular factors. To strengthen the results, VP2-CK2α interaction was validated via co-IP analysis in MSB1 cells infected with CIAV. As shown in Fig. 1C and D, VP2 specifically interacted with CK2α in CIAV-infected cells. Notably, CK2α′ was also detected in the VP2 immunoprecipitates, which may be attributed to the formation of complexes between CK2α and CK2α′ in cells. Furthermore, we analyzed the subcellular localization of VP2 and CK2α in MSB1 cells infected with CIAV. Confocal microscopy revealed co-localization of CK2α with VP2 (Fig. 1E), supporting the conclusion that CIAV VP2 interacts with CK2α in cells following viral infection.

### CIAV infection upregulates CK2α expression in chicken thymus

**Fig 2:**
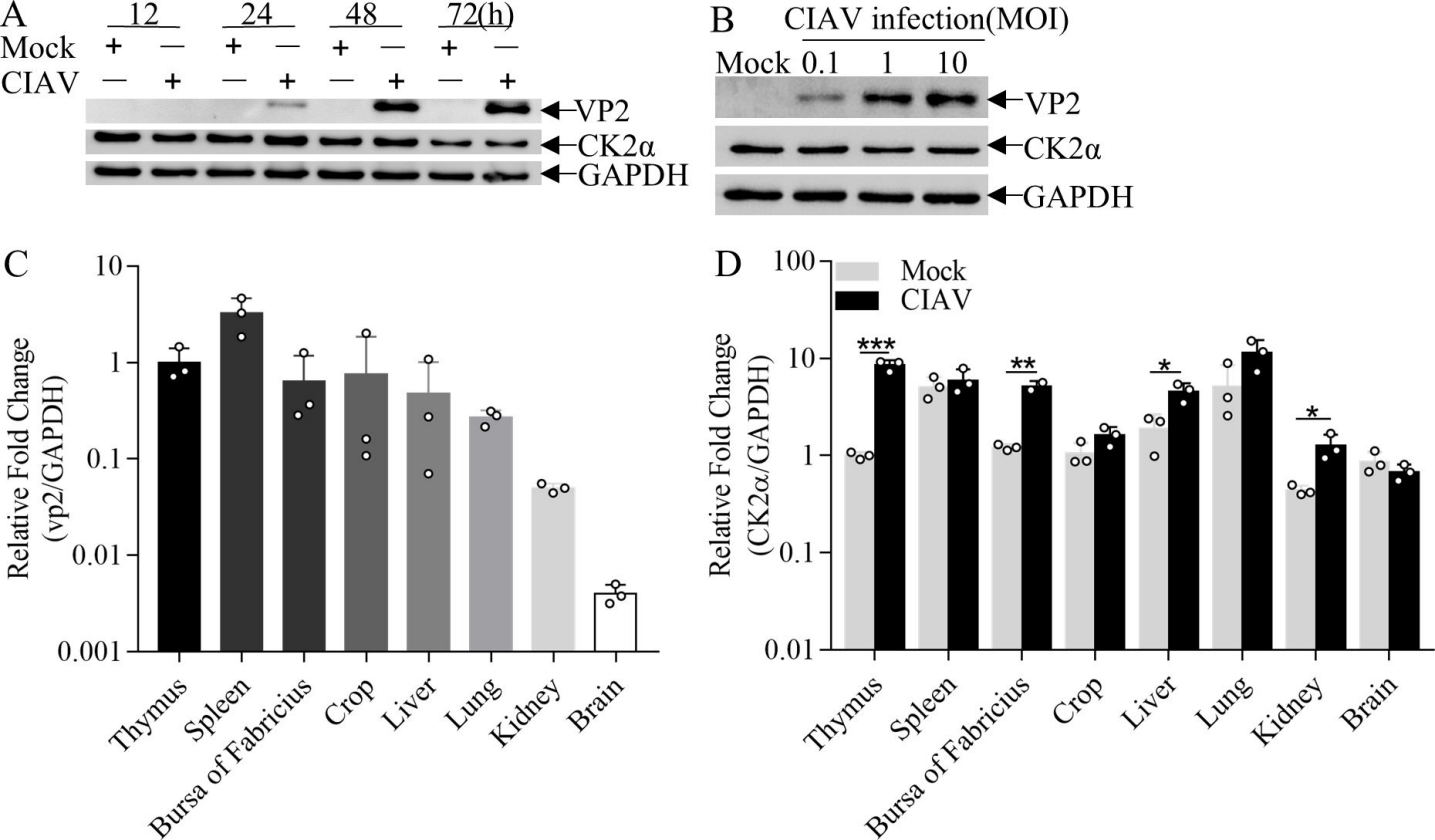
CIAV infection upregulates CK2α expression in chicken thymus tissues. (**A, B**) CK2α expression in CIAV-infected MDCC-MSB1 cells. (**A**) Time-course analysis: MDCC-MSB1 cells were mock-infected or infected with CIAV (MOI = 1), then harvested at 12, 24, 48, and 72 hpi. Western blot analysis was performed using anti-VP2 (viral infection marker), anti-CK2α, and anti-glyceraldehyde-3-phosphate dehydrogenase (GAPDH) (loading control) antibodies. (**B**) Dose-response analysis: MDCC-MSB1 cells were mock-infected or infected with CIAV at the indicated MOIs, then harvested at 24 hpi. Western blot analysis was conducted as described in panel **A**. (**C, D**) Viral VP2 and host CK2α mRNA expression in tissues of CIAV-infected chickens. Mock-infected (*n* = 3) or CIAV-infected (*n* = 3) chickens were euthanized, and tissues from different organs were collected. Total RNA was extracted, and (**C**) VP2 (viral load marker) and (**D**) CK2α mRNA levels were quantified by quantitative PCR (qPCR). GAPDH served as an internal control. Relative mRNA levels were calculated as the ratio of target gene (VP2/CK2α) mRNA to GAPDH mRNA in the same sample. (**C**) VP2 expression was normalized to the VP2 level in the thymus of CIAV-infected chickens (set to 1.0). (**D**) CK2α expression was normalized to the CK2α level in the thymus of mock-infected chickens (set to 1.0). The *y*-axis is presented on a base-10 logarithmic scale to visualize large differences in expression. Data are representative of three independent experiments and presented as means ± standard deviation (SD). Statistical significance: ***, *P* < 0.001; **, *P* < 0.01; *, *P* < 0.05.

In order to assess whether CK2α expression is affected by CIAV infection, CK2α protein levels were examined in MSB1 cells at 12, 24, 48, and 72 h post-infection (hpi) with CIAV at an MOI of 1. The results indicated no significant changes in CK2α protein levels at all time points hpi (Fig. 2A). Also, higher or lower CIAV infection doses (MOI = 0.1 or 10) did not obviously alter CK2α expression either (Fig. 2B). Given that MSB1 cells remain the only established cell line permissive for CIAV replication, we further examined CK2α expression profiles *in vivo*. One-day-old specific pathogen-free (SPF) chickens were intramuscularly injected with CIAV at 10^6^ copies. CK2α mRNA expression level was monitored in the thymus, a primary target of CIAV infection. As shown in Fig. 2C, infection of chickens with CIAV led to a significant expression of the VP2 mRNA in the thymus. Notably, a marked increase in CK2α mRNA expression was observed in the thymus of CIAV-infected chickens compared to mock-infected controls (Fig. 2D). Collectively, these data indicate that CIAV infection does not significantly alter CK2α expression in MSB1 cells *in vitro* but significantly upregulates CK2α mRNA expression in the thymus *in vivo*.

### CK2α facilitates CIAV replication

**Fig 3:**
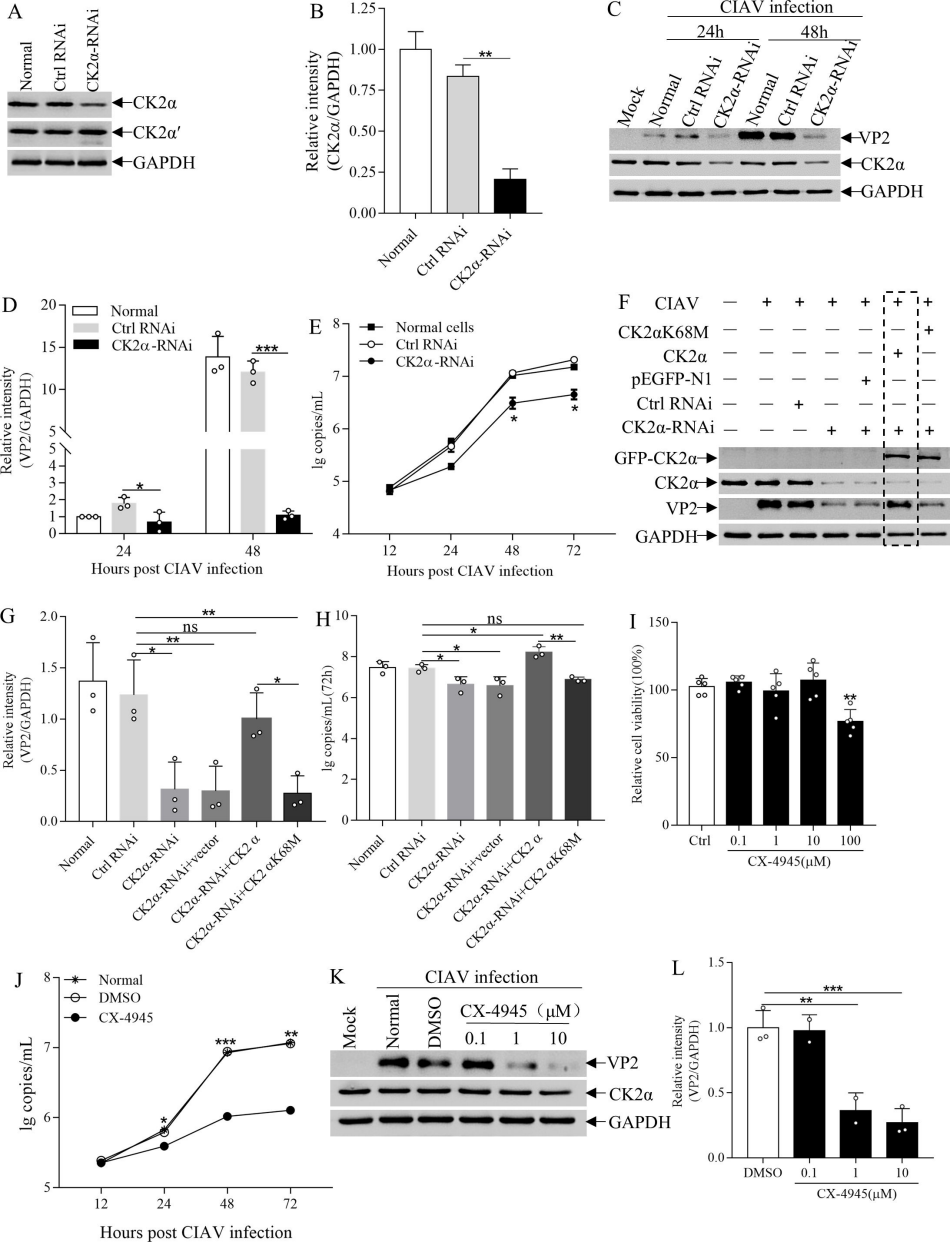
CIAV replication requires CK2α. (**A**) Efficacy of CK2α knockdown by RNAi. MDCC-MSB1 cells were transfected with CK2α-specific siRNA or control siRNA (double transfections at 24 h intervals). At 24 h after the second transfection, cell lysates were analyzed by Western blot using an anti-CK2α antibody. Tubulin served as a loading control. (**B**) Densitometric quantification of CK2α band intensity in panel **A**, normalized to GAPDH. (**C, D**) Effect of CK2α knockdown on CIAV VP2 expression. MDCC-MSB1 cells were transfected with CK2α siRNA or control siRNA, then mock-infected or infected with CIAV (MOI = 1). (**C**) At 24 and 48 hpi, Western blot analysis was performed using anti-VP2, anti-CK2α, and anti-GAPDH antibodies (GAPDH as loading control). (**D**) Densitometric quantification of VP2 bands in panel **C**, expressed as the ratio of VP2 levels in siRNA-transfected cells to those in untreated cells. (**E**) Impact of CK2α knockdown on CIAV growth kinetics. MDCC-MSB1 cells were transfected with CK2α siRNA or control siRNA, then infected with CIAV (MOI = 1). Viral genome copies were quantified by qPCR at 12, 24, 48, and 72 hpi. (F–H) Rescue experiments with wild-type and kinase-inactive CK2α. MDCC-MSB1 cells were transfected with CK2α siRNA (or control siRNA), then transfected with pEGFP-N1-CK2α (wild-type), pEGFP-N1-CK2αK68M (kinase-inactive mutant), or pEGFP-N1 (empty vector) prior to CIAV infection (MOI = 1). (**F**) Western blot analysis at 48 hpi using anti-VP2, anti-CK2α, anti-green fluorescent protein (GFP), and anti-GAPDH antibodies. (**G**) Densitometric quantification of VP2 bands in panel **F**, expressed as the ratio of VP2 levels in transfected cells to those in untreated cells. (**H**) Viral genome copies quantified by qPCR at 72 hpi. (**I**) Cytotoxicity of the CK2α inhibitor CX-4945. MDCC-MSB1 cell viability was assessed by CCK-8 assay 24 h after treatment with CX-4945. (**J**) Effect of CX-4945 on CIAV growth kinetics. MDCC-MSB1 cells were infected with CIAV (MOI = 1), treated with 10 μM CX-4945 2 h post-infection, and viral genome copies were quantified by qPCR at 12, 24, 48, and 72 hpi. (**K, L**) Dose-dependent effect of CX-4945 on VP2 expression. MDCC-MSB1 cells were mock-infected or infected with CIAV (MOI = 1), then treated with CX-4945 (0.1, 1, 10 μM) or dimethyl sulfoxide (DMSO) (control) 2 h post-infection. (**K**) Western blot analysis at 24 hpi using anti-CK2α, anti-VP2, and anti-GAPDH antibodies. (**L**) Densitometric quantification of VP2 bands in panel **K**, expressed as the ratio of VP2 to GAPDH band densities. Data are representative of three independent experiments and presented as means ± SD. Statistical significance: ***, *P* < 0.001; **, *P* < 0.01; *, *P* < 0.05.

Given the fact that CIAV VP2 interacts with CK2α, we further investigated the role of CK2α in CIAV replication via knockdown of CK2α by RNAi. Compared to the control small interfering RNA (siRNA), the siRNA specific for CK2α efficiently reduced CK2α protein expression in MSB1 cells (Fig. 3A and B). Our data also showed that knockdown of CK2α significantly decreased the expression of VP2 in cells with CIAV infection (Fig. 3C and D) and reduced virus titers compared to that of control at 48 and 72 hpi (Fig. 3E). However, overexpression of CK2α did not further enhance CIAV VP2 expression or viral replication in cells with intact CK2α levels (Fig. S1A and B). This result suggests that endogenous CK2α expression may already be sufficient to fully support viral replication. To further determine the role of CK2α in CIAV replication, we investigated whether ectopic expression of CK2α in cells with CK2α knockdown could restore CIAV replication. We expressed EGFP-tagged wild-type CK2α protein in MSB1 cells where CK2α was knocked down by RNAi (Fig. 3F). As expected, the ectopic expression of CK2α was able to restore CIAV VP2 expression (Fig. 3F and G) and virus titer (Fig. 3H). These data indicate that CK2α is required for CIAV replication.

Previous studies have suggested that the kinase activity of CK2α regulates viral replication and that the conserved residue Lys68 (K68) is essential for the enzymatic activity (33, 34). Thus, we investigated whether mutation of CK2α (K68M) would affect CIAV replication. We expressed EGFP-tagged kinase-inactive mutant CK2α (K68M) in MSB1 cells with CK2α knockdown and examined viral replication. Our data showed that expression of mutant CK2α failed to restore CIAV VP2 expression (Fig. 3F and G) and viral replication (Fig. 3H) compared to wild-type CK2α control. These results suggest that the residue Lys68 (K68) of CK2α plays a critical role in CIAV replication.

To strengthen the results, we examined the effect of CX-4945, a selective CK2 (CK2α) inhibitor (35), on CIAV replication. As shown in Fig. 3I, treatment of cells with CX-4945 at 10 μM did not significantly affect cell viability. However, CX-4945 treatment markedly reduced viral growth in time- and dose-dependent manners as compared to that of control (Fig. 3J through L). These results further indicate that CK2α is critical for CIAV replication.

### CK2α kinase stabilizes VP2 through inhibition of proteasomal degradation

**Fig 4:**
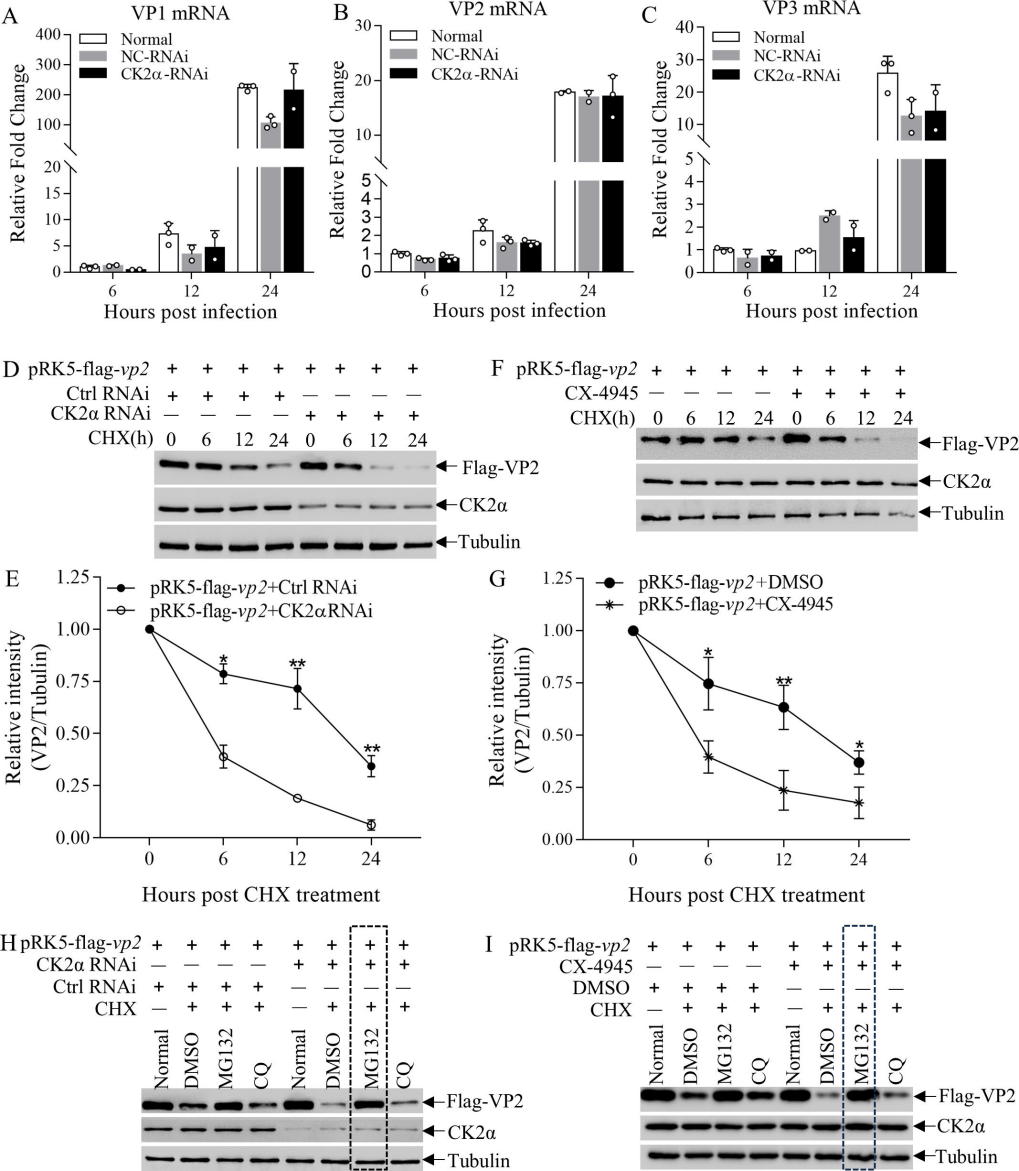
CK2α kinase stabilizes VP2 by inhibiting its proteasomal degradation. (A–C) Effect of CK2α knockdown on CIAV viral gene transcription. MDCC-MSB1 cells were transfected with CK2α-specific siRNA or control siRNA, then infected with CIAV (MOI = 1). At 6, 12, and 24 hpi, qPCR was performed to quantify the mRNA expression levels of VP1 (**A**), VP2 (**B**), and VP3 (**C**). GAPDH served as an internal control. Relative gene expression was calculated as the ratio of viral gene mRNA to GAPDH mRNA at each time point. (**D, E**) VP2 stability under CK2α knockdown. MDCC-MSB1 cells were transfected with CK2α siRNA (or control siRNA) and co-transfected with pRK5-flag-VP2. Twelve hours post-transfection, protein synthesis was inhibited by adding cycloheximide (CHX, 50 µg/mL), and cells were harvested at 0, 6, 12, and 24 h post-CHX treatment. (**D**) Western blot analysis of cell lysates using anti-Flag (for Flag-VP2), anti-CK2α, and anti-Tubulin (loading control) antibodies. (**E**) Densitometric quantification of Flag-VP2 bands in panel **D**, normalized to Tubulin. The normalized Flag-VP2 level at 0 h post-CHX was set to 1.0. (**F, G**) VP2 stability under CK2α kinase inhibition. MDCC-MSB1 cells were pre-treated with 10 μM CX-4945 (CK2α inhibitor) or DMSO (solvent control), then transfected with pRK5-flag-VP2. Twelve hours post-transfection, cells were treated with CHX (50 µg/mL) and harvested at 0, 6, 12, and 24 h post-CHX. (**F**) Western blot analysis using anti-Flag, anti-CK2α, and anti-Tubulin antibodies. (**G**) Densitometric quantification of Flag-VP2 bands in panel **F**, normalized to Tubulin. The normalized Flag-VP2 level at 0 h post-CHX was set to 1.0. (**H**) Effect of degradation pathway inhibitors on VP2 stability during CK2α knockdown. MDCC-MSB1 cells were transfected with CK2α siRNA (or control siRNA) and pRK5-Flag-VP2. Twelve hours post-transfection, cells were treated for 12 h under four conditions: (i) untreated (baseline); (ii) CHX (50 µg/mL) + 0.1% DMSO (protein synthesis inhibition); (iii) CHX + 20 µM MG132 (proteasome inhibition); (iv) CHX + 20 µM chloroquine (lysosome inhibition). Cell lysates were analyzed by Western blot using anti-Flag, anti-CK2α, and anti-Tubulin antibodies. (**I**) Effect of degradation pathway inhibitors on VP2 stability during CK2α inhibition. MDCC-MSB1 cells were pre-treated with DMSO or CX-4945, then transfected with pRK5-Flag-VP2. Twelve hours post-transfection, cells were treated under the same four conditions as in panel **H**. Western blot analysis was performed using anti-Flag, anti-CK2α, and anti-Tubulin antibodies. Data are representative of three independent experiments and presented as means ± SD. Statistical significance: ***, *P* < 0.001; **, *P* < 0.01; *, *P* < 0.05.

Since CK2α was important for CIAV replication, we further explored the underlying mechanism. We first examined the effect of CK2α knockdown on CIAV mRNA transcription, and the results indicated that the mRNA levels of VP1, VP2, and VP3 remained unaffected by CK2α knockdown (Fig. 4A through C). Then, we assessed the impact of CK2α on VP2 protein stability by transfecting MSB1 cells with CK2α-targeting siRNA or control siRNA and subsequently with Flag-tagged VP2. Notably, CK2α knockdown significantly accelerated the degradation of VP2 (Fig. 4D and E). Consistent with this observation, pharmacological inhibition of CK2α kinase activity by CX-4945 (10 μM) similarly enhanced VP2 degradation (Fig. 4F and G), indicating that CK2α is essential for maintaining VP2 stability. To identify the degradation pathway of VP2, CHX-treated cells were co-incubated with either MG132 (a proteasome inhibitor) or chloroquine (CQ, a lysosome inhibitor). Our data showed that VP2 degradation was significantly blocked by MG132 rather than by CQ (Fig. 4H and I), suggesting that VP2 is primarily degraded via the proteasomal pathway. Taken together, these findings imply that CK2α is essential for VP2 stabilization.

### Ser182 and Asp183 of VP2 are critical for VP2-CK2α interaction

**Fig 5:**
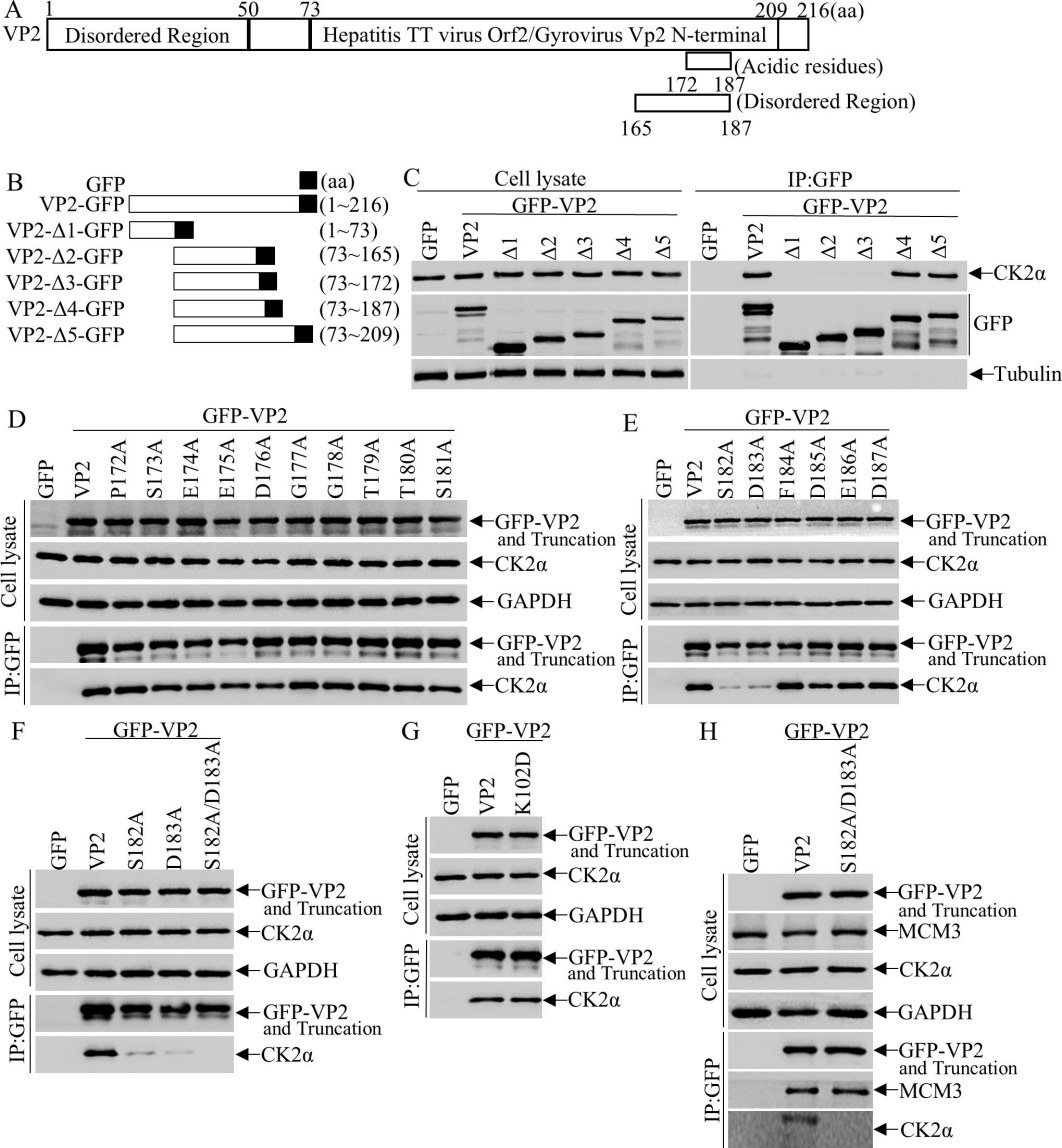
Ser182 and Asp183 of CIAV VP2 are critical for VP2-CK2α interaction. (**A**) Schematic representation of the full-length CIAV VP2 protein (amino acid [aa] positions indicated). (**B**) Schematic diagrams of the genes encoding full-length VP2 and its truncated mutants (Δ1 to Δ5). The amino acid ranges of each truncation are labeled to indicate deleted regions relative to full-length VP2. (**C–H**) Mapping of the CK2α-binding domain in VP2 using truncated mutants. DF-1 cells were transfected with pEGFP-N1-VP2 (full-length VP2), pEGFP-N1-VP2 truncated mutants, or empty pEGFP-N1 vector (negative control). At 24 h post-transfection, cell lysates were subjected to immunoprecipitation (IP) using an anti-GFP monoclonal antibody (to pull down EGFP-tagged VP2/fragments). IP pellets were analyzed by Western blot with: (i) anti-GFP antibody (to detect EGFP-VP2/fragments), (ii) anti-CK2α antibody (to detect co-immunoprecipitated CK2α), and (iii) anti-Tubulin or anti-GAPDH antibody (loading control for input lysates). Input lysates (prior to IP) were also probed to confirm expression of EGFP-VP2/fragments and endogenous CK2α.（**C**) VP2 truncation mutants Δ1–Δ5.(**D, E**) VP2 single-point mutations within aa 172–187. (**F**) VP2 single-point mutations S182A and D183A, and the double mutation S182A/D183A.(G)VP2 K102D mutation.(H) VP2 double mutation S182A/D183A.Ser182 and Asp183 of CIAV VP2 are critical for VP2-CK2α interaction.

To further identify the key amino acid (aa) residues of VP2 involved in VP2-CK2α interaction, we constructed a series of VP2 truncation mutants (Δ1–Δ5) based on the predicted topology of VP2 (Fig. 5A and B) and evaluated their capacity to bind CK2α using co-IP assays. Our data showed that mutants Δ1–Δ3, which lack the sequence 172–187 aa, failed to bind CK2α (Fig. 5C), whereas the full-length of VP2 as well as mutants Δ4 and Δ5 interacted with CK2α, indicating that residues 172–187 (aa 172–187) are indispensable for VP2-CK2α interaction. To identify the specific residues within this region critical for binding, we conducted a systematic alanine-scanning mutagenesis analysis by substituting each amino acid in the 172–187 segment of VP2 with alanine. As shown in Fig. 5D and E, individual mutation at Ser182 (S182A) or Asp183 (D183A) significantly impaired the VP2-CK2α interaction, whereas mutations at other positions within this segment exerted negligible effects on binding. We therefore prioritized these two residues for in-depth characterization of their role in the VP2-CK2α interaction. Co-IP assays confirmed that introduction of the S182A/D183A double mutation completely abrogated the VP2-CK2α interaction (Fig. 5F). VP2 K102D mutation has been reported to impair viral replication (21). To verify the specificity of the S182A/D183A mutation, we used VP2 K102D mutant as a control and found that mutation at VP2 K102D did not affect the ability of VP2 to bind CK2α (Fig. 5G), indicating that this site is not involved in the CK2α interaction. Moreover, as shown in Fig. 5H, the VP2 S182A/D183A mutant maintained its interaction with another established binding partner, MCM3 (36), demonstrating that the mutation does not cause a complete disruption of VP2 structure. These data suggest that the Ser182 and Asp183 of VP2 are specifically required for the VP2-CK2α interaction.

### Double mutations at S182A and D183A of VP2 impair CIAV replication

**Fig 6:**
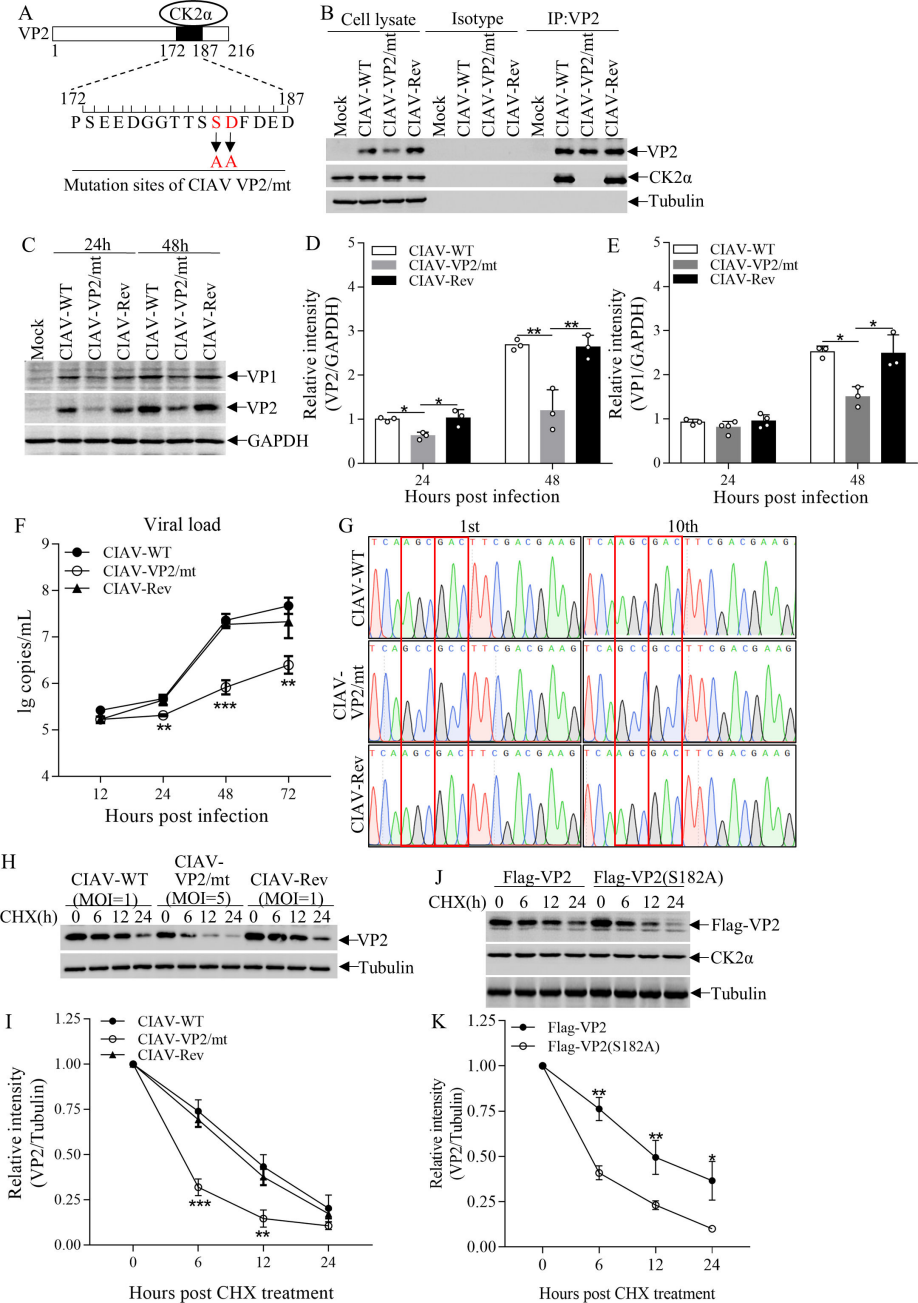
Double mutations S182A and D183A of VP2 impaired CIAV replication. (**A**) Schematic diagram of CIAV VP2 highlighting the amino acid mutation sites: Ser182 (S182) and Asp183 (D183) (mutated to alanine in the VP2 mutant, denoted as “mt”). (**B**) Effect of VP2 mutations on interaction with endogenous CK2α. MDCC-MSB1 cells were mock-infected or infected with CIAV-WT, CIAV-VP2/mt, or CIAV-Revertant (Rev) (MOI = 1). At 48 hpi, co-IP was performed using an anti-VP2 mAb. The quantity of antibody-conjugated beads was optimized to ensure saturation of binding sites, allowing for comparable immunoprecipitation efficiency across samples. Co-immunoprecipitated CK2α was detected by Western blot using an anti-CK2α antibody. “Isotype” refers to the IgG1 isotype control antibody for the anti-VP2 mAb. Input lysates were probed to confirm expression of VP2 and CK2α. (C–E) Viral protein expression in cells infected with CIAV-VP2/mt. MDCC-MSB1 cells were infected with CIAV-WT, CIAV-VP2/mt, or CIAV-Rev (MOI = 1). (**C**) At 24 and 48 hpi, Western blot analysis of cell lysates was performed using anti-VP1, anti-VP2, and anti-GAPDH (loading control) antibodies. Densitometric quantification of (**D**) VP1 and (**E**) VP2 band intensities in panel **C**, normalized to GAPDH. (**F**) Growth kinetics of CIAV VP2/mt. MDCC-MSB1 cells were infected with CIAV-WT, CIAV-VP2/mt, or CIAV-Rev (MOI = 1). Viral genome copies were quantified by qPCR at 12, 24, 48, and 72 hpi. (**G**) Genetic stability of VP2-targeted mutations. CIAV-VP2/mt was serially passaged 10 times in MDCC-MSB1 cells. Viral genomic DNA was extracted, and the VP2 mutation region was amplified by PCR. Ten single clones were subjected to Sanger sequencing to verify the stability of S182/D183 mutations. (**H, I**) VP2 stability in cells infected with CIAV VP2/mt. MDCC-MSB1 cells were infected with CIAV-WT, CIAV-VP2/mt, or CIAV-Rev. At 24 hpi, protein synthesis was inhibited by CHX (50 µg/mL), and cells were harvested at 0, 6, 12, and 24 h post-CHX treatment. (**H**) Western blot analysis of cell lysates using anti-VP2 and anti-Tubulin (loading control) antibodies. (**I**) Densitometric quantification of VP2 band intensities in panel **H**, normalized to Tubulin. The normalized VP2 level at 0 h post-CHX was set to 1.0. (**J, K**) VP2 stability of the S182A phospho-deficient mutant. MDCC-MSB1 cells were transfected with pRK5-Flag-VP2 or pRK5-Flag-VP2(S182A). Twelve hours post-transfection, protein synthesis was inhibited by CHX (50 µg/mL), and cells were harvested at 0, 6, 12, and 24 h post-CHX treatment. (**J**) Western blot analysis of cell lysates using anti-Flag, anti-CK2α, and anti-Tubulin (loading control) antibodies. (**K**) Densitometric quantification of Flag-VP2 band intensities in panel **J**, normalized to Tubulin. The normalized level of each protein at 0 h post-CHX was set to 1.0. Data are representative of three independent experiments and presented as means ± SD. Statistical significance: ***, *P* < 0.001; **, *P* < 0.01; *, *P* < 0.05. “mt” = CIAV-VP2/mutant; “Rev” = CIAV-Revertant.

To evaluate the role of the VP2-CK2α interaction in viral replication, we constructed a recombinant CIAV with VP2 S182A/D183A (CIAV-VP2/mutant) (Fig. 6A) and a recombinant CIAV carrying VP2 S182/D183 revertant mutation (CIAV-Revertant) as a control. As shown in Fig. 6B, endogenous CK2α could be precipitated with VP2 in cells infected with either CIAV-WT or CIAV-Revertant, but not with CIAV-VP2/mutant. Importantly, we found that the expression levels of VP1 and VP2 in cells with CIAV-VP2/mutant infection were markedly reduced compared to those infected with CIAV-WT or CIAV-Revertant at 24 and 48 hpi (Fig. 6C through E). Furthermore, the viral loads of CIAV-VP2/mutant in cell cultures were significantly lower than those of CIAV-WT or CIAV-Revertant at 24, 48, and 72 hpi (Fig. 6F). These data demonstrate that the VP2 S182A/D183A double mutant impairs CIAV replication, highlighting the critical role of VP2-CK2α interaction in viral replication.

To assess the genetic stability of CIAV-VP2/mutant, we sequenced the 1st and 10th generations of the virus, focusing on the VP2 mutation region, with CIAV-WT and CIAV-Revertant serving as controls. As shown in Fig. 6G, the sequences at the two mutation sites remained unaltered in both the 1st and 10th generations of CIAV-VP2/mutant. These results indicate that the targeted mutation region in CIAV-VP2/mutant exhibits high genetic stability. Given that the VP2-CK2α interaction is essential for VP2 stability and the VP2 S182A/D183A double mutation blocks the interaction, we further determined the effect of these mutations on VP2 stability in CIAV-infected cells. Our data showed that, in comparison with CIAV-WT or CIAV-Revertant infection, CIAV-VP2/mutant infection significantly increased VP2 degradation (Fig. 6H and I), confirming the role of VP2-CK2α interaction in viral replication under physiological conditions. To further pinpoint the critical residue, we constructed the VP2 S182A phospho-deficient mutant and analyzed its protein stability. Consistent with the double mutant, the VP2 S182A single mutant exhibited significantly faster degradation compared to wild-type VP2 (Fig. 6J and K). These results indicate that VP2 mutations (S182A/D183A) not only reduce CIAV replication but also affect VP2 protein stability, most likely by disrupting its interaction with CK2α, thus confirming the essential role of VP2-CK2α interaction in sustaining viral replication.

### The VP2 S182A/D183A double mutations attenuate the virulence of CIAV

**Fig 7:**
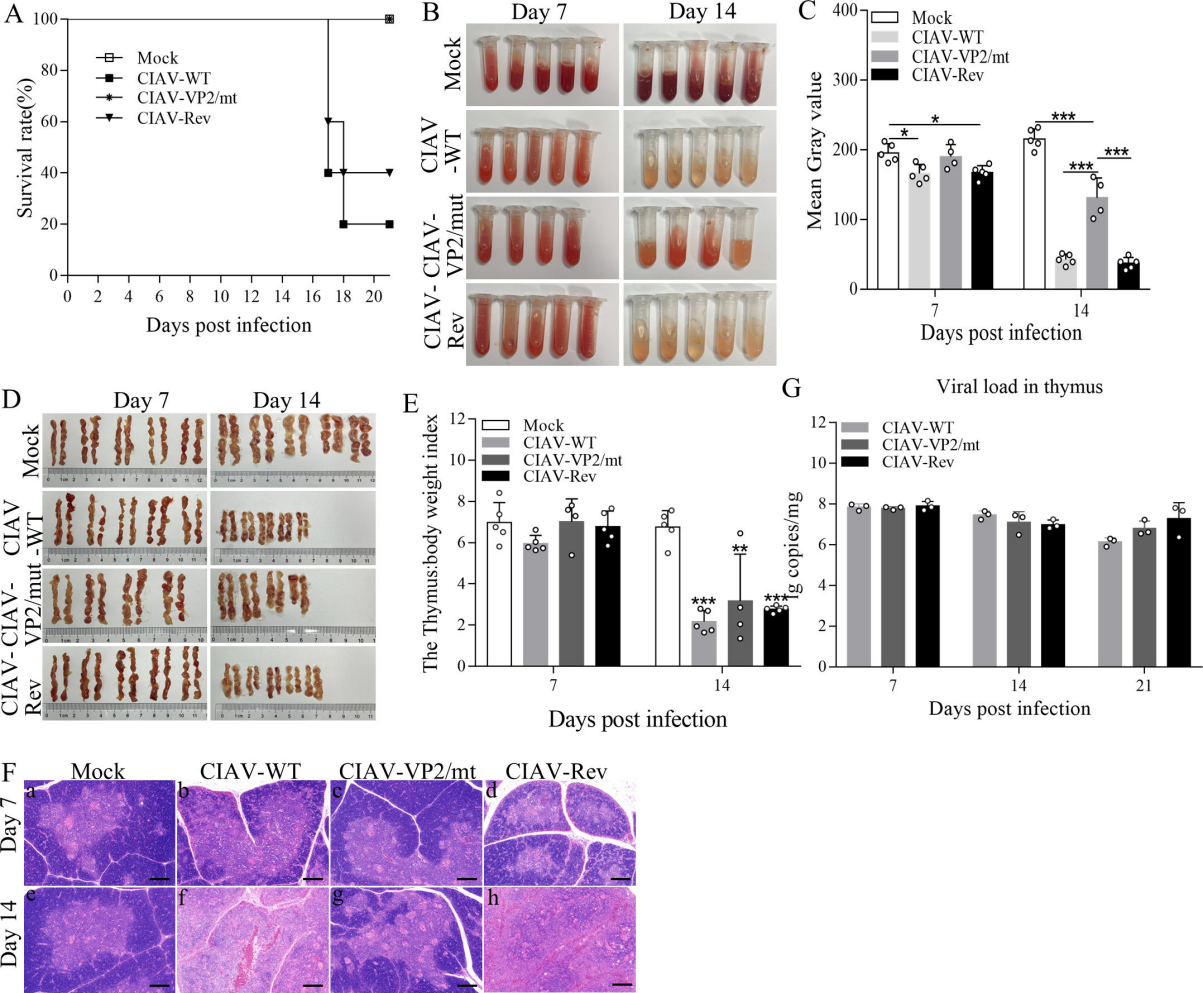
The VP2 S182A/D183A double mutations attenuated the virulence of CIAV. (**A**) Survival rates of chickens inoculated with CIAV-WT, CIAV-VP2/mt, CIAV-Rev, or mock-inoculated (RPMI 1640) from 1 to 21 days post-inoculation (dpi) (*n* = 5 per group). (**B, C**) Assessment of anemia via bone marrow lavage fluid (BMLF) analysis. (**B**) Representative images of chicken BMLF at 7 and 14 dpi; (**C**) Mean gray value quantification of BMLF samples in panel **B** (an indicator of anemia severity, with lower gray values reflecting more severe anemia). (**D, E**) Thymus damage evaluation. (**D**) Representative gross morphology of chicken thymus at 7 and 14 dpi; (**E**) thymus-to-body weight ratio calculated at 7 and 14 dpi (a measure of thymus atrophy, with lower ratios indicating more severe atrophy). (**F**) Histopathological analysis of thymus tissues (hematoxylin and eosin [H&E] staining). Thymus samples were fixed, sectioned, and stained at 7 and 14 dpi. Scale bar = 200 µm. 7 dpi: (a) Mock group: no visible lesions, with distinct demarcation between thymic cortex and medulla; (b) CIAV-WT group: severe cortical atrophy and blurred cortex-medulla boundaries; (c) CIAV-VP2/mt group: no obvious lesions, with clear cortex-medulla demarcation; (d) CIAV-Rev group: similar lesions to WT (cortical atrophy, blurred boundaries). 14 dpi: (e) Mock group: no lesions; (f) CIAV-WT group: significant thymic hemorrhage and cortical atrophy; (g) CIAV-VP2/mt group: mild cortical atrophy (less severe than WT/Rev); (h) CIAV-Rev group: severe hemorrhage and cortical atrophy (similar to WT). (**G**) Viral replication in thymus tissues. Thymus samples were collected and homogenized at 7 and 14 dpi. Viral genomic DNA was extracted, and viral copy numbers were quantified by qPCR (targeting the VP1 gene). Data are representative of four to five independent samples (*n* = 4–5) and presented as means ± SD. Statistical significance: ***, *P* < 0.001; **, *P* < 0.01; *, *P* < 0.05. “mt” = mutant; “Rev” = Revertant.

Since the VP2 S182A/D183A double mutation impairs CIAV replication in host cells, we hypothesize that infection of chickens with the CIAV-VP2/mutant would reduce viral pathogenicity. To test this hypothesis, 1-day-old SPF chickens were intramuscularly injected with CIAV-VP2/mutant, CIAV-WT, or CIAV-Revertant at a dose of 10^6^ copies of virus in 100 μL per chicken, and the mock controls received an equal volume of Roswell Park Memorial Institute 1640 (RPMI 1640) via the same route. As shown in Fig. 7A, more than half of the chickens (*n* = 5/group) succumbed to death at 17 and 18 days post-infection with CIAV-WT and CIAV-Revertant. In contrast, all chickens infected with CIAV-VP2/mutant survived throughout the experiment. Furthermore, the bone marrow displayed severe hypoplasia in CIAV-WT and CIAV-Revertant infected chickens 14 days post-infection, characterized by pale and watery features. These lesions were markedly ameliorated in chickens infected with the CIAV-VP2/mutant (Fig. 7B and C). These data indicate that VP2 S182A/D183A mutations significantly attenuate CIAV virulence in chickens.

The thymus serves as the site for T lymphocyte development and maturation, and its impairment leads to immunosuppression. As infection of chickens with CIAV causes damage to the thymus (9, 37), we examined the pathological changes in the thymus of chickens at 7 and 14 days post-infection with CIAV. We found that there were no differences in gross lesions between chickens infected with different strains of CIAV and mock controls at 7 days post-infection (Fig. 7D and E). In contrast, all CIAV-infected groups exhibited marked thymic atrophy, as demonstrated by thymus-to-body weight ratio at 14 days post-infection. However, histopathological examination showed that infection of chickens with recombinant CIAV VP2 S182A/D183A did not cause obvious changes in the thymus, whereas thymuses from CIAV-WT and CIAV-Revertant-infected chickens displayed cortical atrophy and loss of the distinct corticomedullary junction (Fig. 7F). Notably, viral genome quantification revealed comparable levels of viral replication in the thymus of all infected chickens at different time points (Fig. 7G). These results indicate that CIAV VP2 S182/D183 plays a critical role in the pathogenesis of CIAV infection.

## DISCUSSION

CIAV remains a severe threat to global poultry production, causing severe immunosuppression and aplastic anemia in chickens (2, 38). The infected chickens display high mortality, stunted growth, and increased susceptibility to secondary infections (37, 39, 40), leading to substantial economic losses. The CIAV infection in vaccinated flocks usually persists due to viral evolution and incomplete cross-protection (41, 42). Given that VP2 plays multifaceted roles in viral assembly, phosphatase activity, and modulation of host innate immunity, particularly in the cGAS-STING pathway, there is a compelling need to elucidate how VP2 exploits host machinery to facilitate viral replication. The present study provides novel evidence that CK2α functions as a proviral factor by stabilizing CIAV VP2 protein. The consistent results from siRNA knockdown and CX-4945 inhibition demonstrate that both CK2α expression and kinase activity are indispensable for VP2 stability and viral replication.

CK2α, the catalytic subunit of casein kinase II, is a highly conserved serine/threonine kinase involved in diverse cellular processes, including signal transduction, transcription regulation, and cell cycle. Our data indicate that CK2α prevents CIAV VP2 from proteasomal degradation by interacting with it. This observation is consistent with previous findings on the role of CK2α in enhancing protein stability. It has been reported that CK2α phosphorylates glucose-regulated protein 94 at Ser306, increasing its stability and interaction with LRP6 to activate Wnt signaling during triple-negative breast cancer metastasis (43). Similarly, CK2α regulates HPV E1 protein stability and nuclear localization via kinase-dependent mechanisms to promote viral genome replication (44). Notably, CK2-mediated phosphorylation of Thr242 in Potato virus A capsid protein promotes infection, with this residue embedded in the 242-TTSEED-247 motif conforming to the CK2 consensus (S/T)XX(D/E) (45). In this study, we identified Ser182 and Asp183 of VP2 as essential residues for its interaction with CK2α. Notably, the local sequence context surrounding Ser182 (residues 180–183: Thr180-Ser181-Ser182-Asp183) closely matches the CK2 substrate consensus motif (S/T)XX(D/E), where Thr180 occupies the (S/T) position, Ser182 occupies a central X position, and Asp183 occupies the critical acidic (D/E) position. This sequence alignment strongly suggests that Ser182 (and possibly the adjacent Thr180) represents a putative CK2 phosphorylation site integral to the interaction interface. While Asp183 itself is not a phosphor-acceptor residue, its presence as the defining acidic residue in the consensus motif is crucial for substrate recognition by CK2. Attempts to directly confirm CK2-mediated phosphorylation at Ser182 via phospho-proteomics are unfortunately hampered by technical limitations, including potential low stoichiometry of phosphorylation, peptide coverage issues, or dynamic modification states inherent to viral proteins during infection, preventing definitive assignment. Nevertheless, the functional importance of Ser182 and Asp183 for the VP2-CK2α interaction and the detrimental effects of their mutation on VP2 stability and viral replication strongly suggest that CK2α binding is essential for VP2 stability.

Furthermore, our *in vivo* experiments revealed significant upregulation of CK2α in the thymus of CIAV-infected chickens. The thymus is the primary viral target organ and a central immune organ in chickens. This tissue-specific increase in CK2α expression highlights the physiological significance of CK2α during viral infection and strongly suggests that CIAV actively manipulates the host environment to ensure sufficient CK2α availability for its replication. Our data demonstrate CIAV’s dependence on CK2α for its replication, underscoring the heavy reliance of the virus on cellular factors for its survival. Numerous viruses have been reported to exploit kinases or signaling cascades to stabilize viral proteins, evading immune responses or enhancing replication efficiency. For instance, Marek’s disease virus, an oncogenic alpha herpesvirus, encodes the serine/threonine kinase Us3, which phosphorylates and inhibits the nuclear translocation of interferon regulatory factor 7, thereby suppressing IFN-β production and enhancing viral replication (46). Similarly, among circoviruses, porcine circovirus type 2 has been shown to induce autophagy and promote viral replication by inhibiting mTOR through a phosphorylated protein cascade involving ERK1/2 and AMPK (47). CK2 has been shown to be upregulated in SARS-CoV-2 infection, forming a complex with the viral capsid protein (31, 48). The broad ability of CX-4945 to disrupt replication of both β-coronaviruses and CIAV underscores its potential as a broad-spectrum host-targeted antiviral drug, especially against CK2-dependent viruses. CK2α is known to phosphorylate proapoptotic proteins such as BID at Thr58 and Ser76, thereby inhibiting caspase-8 cleavage and mitochondrial apoptosis (49). By suppressing apoptosis, this mechanism likely prolongs host cell survival, providing a sustained environment conducive to CIAV replication. Moreover, CK2α plays a direct role in DNA replication and cell cycle progression by maintaining the expression of the mini-chromosome maintenance (MCM) protein complex, which is critical for S-phase progression (50). Structurally optimized bivalent inhibitors based on CX-4945 have recently been developed and shown to potently inhibit β-coronavirus (including SARS-CoV-2) replication *in vitro* by disrupting CK2-dependent processes essential for viral hijacking of host cells (51). Consistent with this broader role of CK2 in viral infections, our present study demonstrates that the selective CK2 inhibitor CX-4945 also potently suppresses CIAV replication *in vitro*. These collective findings highlight the expansive role of CK2α in viral and cellular biology. Consequently, there is a strong rationale for further investigation into the *in vivo* efficacy, safety, and spectrum of activity of CX-4945 against other CK2-dependent viruses. Such studies, particularly in veterinary contexts (e.g., against CIAV), hold significant promise for practical application.

It is noteworthy that CIAV VP2 has been identified as a chromatin-binding protein that interacts with MCM3 in cells (36). This interaction raises the possibility that CIAV could exploit host DNA replication machinery to divert cellular resources toward viral replication. Moreover, CK2α-enhanced PI3K/Akt and NF-κB signaling pathways are often manipulated by viruses to suppress host antiviral responses (52, 53). In the context of CIAV infection, CK2α-mediated stabilization of VP2 might indirectly disrupt host immune surveillance, although this requires experimental validation. These mechanistic parallels highlight CK2α’s evolutionary versatility as a kinase exploited by pathogens to manipulate host processes.

There is growing evidence that targeted mutagenesis of CIAV proteins has a profound effect on viral pathogenicity. It has been found that mutating glutamic acid to histidine at position 394 of VP1 protein reduces the pathogenicity of CIAV (54). It is predicted that amino acids 75, 125, 141, 144, and 287 of VP1 are associated with viral virulence by selection pressure analysis (55). Mutation of threonine to alanine at positions 56 and 61 of VP3 significantly inhibits CIAV virus replication (56). The functional importance of VP2 is highlighted by studies of specific point mutations: alterations within the DSP catalytic motif (e.g., K102D, D161G/E162G) or in a predicted structured region (e.g., R101G) are found to substantially reduce viral replication or attenuate viral pathogenicity (21). In the present study, we reveal a previously unrecognized mechanism by which CIAV exploits host kinase activity to enhance its replication, through a specific interaction between VP2 and CK2α, with Ser182 and Asp183 of VP2 identified as key amino acids for this binding. Through this interaction, CIAV hijacks the host kinase activity to stabilize its key nonstructural protein VP2. Importantly, our data showed that unlike mutations targeting the catalytic motif or predicted structural domain of VP2, the S182A/D183A double mutations markedly shortened VP2 half-life and affected its stability. Furthermore, we successfully constructed a mutant CIAV harboring these double mutations, which exhibited significantly attenuated pathogenicity. Collectively, these findings demonstrate that disrupting the VP2-CK2α interaction reduced viral replication and virulence.

In conclusion, our data demonstrate that host kinase CK2α promotes CIAV infection by stabilizing the viral VP2 protein, and disruption of the VP2-CK2α interaction impairs viral replication. Furthermore, mutation of VP2 at sites S182 and D183 markedly attenuated viral pathogenesis. These findings advance our understanding of CIAV pathogenesis and provide a rationale for developing novel intervention strategies targeting the VP2-CK2α interaction for the prevention and control of CIAV infection.

## MATERIALS AND METHODS

### Cells and virus

DF-1 cells (immortal chicken embryo fibroblasts) were obtained from the American Type Culture Collection and cultured in Dulbecco’s modified Eagle medium (Gibco, USA) supplemented with 10% fetal bovine serum (FBS; Sigma-Aldrich, USA) in a 5% CO_2_ incubator at 37°C. MDCC-MSB1 cells were cultured in RPMI 1640 (Macgene Biotechnology, Beijing, China) supplemented with 10% FBS in a 5% CO_2_ incubator at 37°C. CIAV Cux-1 strain (GenBank accession number M55918.1) was kindly provided by Professor Bing Yang (Institute of Animal Husbandry and Veterinary Medicine, Beijing Academy of Agricultural and Forestry Sciences, Beijing, China).

### Animals

One-day-old SPF white Leghorn chicks were provided by Beijing Boehringer Ingelheim Vital Biotechnology Limited Company (Beijing, China). Following inoculation, chickens were housed in separate isolators with *ad libitum* access to feed and water.

### Reagents

Mouse monoclonal antibody against CIAV VP2 was purchased from SAE Biomed-tech (ZS) Company (China). Anti-CK2α (CSNK2A1; A19683) antibody was obtained from ABclonal Technology (Wuhan, China). Anti-FLAG mouse monoclonal antibody (F1804) was obtained from Sigma-Aldrich (USA). Anti-GAPDH (60004-1-Ig) was obtained from Proteintech (Wuhan, China). Anti-α-tubulin (PM054) rabbit antibodies were obtained from Medical Biological Laboratories (Japan). Anti-GFP rabbit polyclonal antibody (66002-1-Ig) and GFP-Trap Agarose were obtained from Proteintech (Wuhan, China). TRITC-conjugated goat anti-rabbit IgG, FITC-conjugated goat anti-mouse IgG, and horseradish peroxidase-conjugated goat anti-mouse and anti-rabbit IgG antibodies were purchased from Ding Guo Chang Sheng Company (Beijing, China). DAPI was purchased from Solarbio Life Sciences (Beijing, China). Mouse IgG isotype control antibody (sc-2025) was purchased from Santa Cruz Biotechnology (USA). Protein A/G Plus-Agarose was purchased from Cytiva (formerly GE Healthcare Life Sciences; Uppsala, Sweden). The pEGFP-N1 vector and pRK5-Flag plasmid were obtained from Takara Bio USA, Inc. (Mountain View, CA, USA). Restriction enzymes were purchased from New England Biolabs, Inc. (Ipswich, MA, USA). QIAfilter Plasmid Midi Kits were purchased from QIAGEN GmbH (Hilden, Germany). The CK2 inhibitor CX-4945 (Silmitasertib) was purchased from APExBIO Technology LLC (Houston, TX, USA). Lipofectamine 3000 transfection reagent was obtained from Thermo Fisher Scientific Inc. (Waltham, MA, USA). CHX (HY-12320), MG132 (HY-13259), CQ (HY-17589A), and protease inhibitor cocktail (HY-K0010) were purchased from MedChemExpress (China).

### Plasmid construction

Chicken CK2α (GenBank accession number NM_001002242.2) coding sequence was amplified by PCR from MDCC-MSB1 cells using the following specific primers: sense primer 5′-ATGTCGGGACCCGTGCCGAGCAGGG-3′ and antisense primer 5′-CTGCTGAGCGCCTGCAGCAGCTGGA-3′. The primers were synthesized by Sangon Biotech Co., Ltd. (Shanghai, China). The pEGFP-N1-CK2α plasmid was constructed using standard molecular biology techniques. The CK2α-K68M mutant was generated by overlap extension PCR using forward primer 5′-TGTTATGATTCTCAAGCCTGTTAAAAAGAAGAA-3′ and reverse primer 5′-GGCTTGAGAATCATAACAACTACTTTTTCATTATTTGTAATGTTG-3′.

### Transfection of MDCC-MSB1 cells

MDCC-MSB1 cells in log phase were harvested and washed twice with Opti-MEM I Reduced Serum Medium, and 2 × 10^6^ cells were resuspended in 100 μL Opti-MEM containing either 10 μg of plasmid DNA or siRNA at a final concentration of 100 nM in a 1.5 mL Eppendorf tube. After gentle pipette mixing, the mixture was transferred to a 0.4 cm gap electroporation cuvette. Transfection was performed using X-Porator H1 (Etta Biotech, China) under the following parameters: 200 V operating voltage, 1400 μs pulse duration, 4 pulses, and 635 ms interval between pulses.

### Knockdown of CK2α by RNAi

The siRNA were designed and synthesized by Genepharma Company (Suzhou, China) and used to knock down the expression of CK2α in MDCC-MSB1 cells. The sense sequence of siRNA targeting CK2α in MDCC-MSB1 cells was as follows: 5′-GCACAGAAAGCUUAGACUATT-3′. MDCC-MSB1 cells were cultured in 12-well plates for 12 h before transfection with siRNA or controls. Double transfections were performed at 24 h intervals. Twenty-four hours after the second transfection, cells were harvested for subsequent analysis.

### Pull-down assay

MDCC-MSB1 cells were seeded in T175 cell culture flasks before infection. At 48 h post-infection, cell lysates were prepared using a lysis buffer (50 mM Tris-HCl, pH 8.0, 150 mM NaCl, 5 mM EDTA, 1% NP-40, 10% glycerol, 1× complete cocktail protease inhibitor). The cell lysates were centrifuged at 12,000 r/min for 10 min at 4°C, and the supernatants were collected. For the immunoprecipitation procedure, the supernatants were first incubated with 10 μg of normal mouse IgG and 60 μL of Protein A/G Plus Agarose beads at 4°C for 12 h. After this pre-clearing step, the beads were removed by centrifugation, and the supernatants were then incubated with 10 μg of anti-VP2 antibody and fresh Protein A/G Plus Agarose beads (60 μL) at 4°C for an additional 12 h. Beads were then washed six times with lysis buffer and boiled with 2*×* SDS loading buffer for 10 min. After centrifugation, the supernatants were subjected to 12% SDS-PAGE and stained with Coomassie Brilliant Blue R-250. After separation of proteins on SDS-PAGE gel, a protein band of approximately 40 kDa, which was specifically enriched in the VP2 sample but absent in the control lane, was excised from the gel. The differential band was subjected to in-gel trypsin digestion followed by LC-MS/MS analysis to identify the specific interacting partner.

### Western blot analysis

Cell lysates were prepared as previously described. Protein samples were boiled with 1× SDS loading buffer for 10 min. Equal amounts of protein were resolved by SDS-PAGE and transferred onto polyvinylidene difluoride membranes. After blocking with 5% (wt/vol) skimmed milk to prevent nonspecific binding, the membranes were incubated overnight at 4°C with the indicated primary antibodies. Protein bands were detected using an enhanced chemiluminescence substrate and visualized with a chemiluminescence imaging system.

### GST pull-down assay

Recombinant plasmids pET-28a-His-VP2 and pGEX-6P-1-CK2α were individually transformed into *E. coli* BL21(DE3) competent cells. Protein expression was induced with 1 mM isopropyl β-D-1-thiogalactopyranoside for 20 h at 16°C. Bacterial cultures were harvested by centrifugation, resuspended in PBS, and lysed by sonication. The lysates were clarified by centrifugation, and the supernatants containing GST-CK2α fusion protein were incubated with Glutathione Sepharose 4B beads (Cytiva Lifesciences; Marlborough, MA, USA), while those containing His-VP2 were incubated with High Affinity Ni-Charged Resin FF (GenScript; Nanjing, China) overnight at 4°C. After five washes with PBS, the recombinant proteins were eluted and analyzed by SDS-PAGE to assess purity. For the pull-down assay, 10 μg of purified GST-CK2α was immobilized onto 50 μL of Glutathione Sepharose 4B beads overnight at 4°C. After washing the beads five times with PBS, 10 μg of purified His-VP2 was added and incubated at room temperature for 2 h. Following five additional washes with PBS, the bound protein complexes were eluted and analyzed by Western blot. Purified GST protein served as a negative control.

### Immunoprecipitation

For the immunoprecipitation of viral VP2 with endogenous CK2α, MDCC-MSB1 cells were mock-infected or infected with CIAV at an MOI of 1. Forty-eight hours after infection, the cell lysates were prepared and subjected to immunoprecipitation with an anti-VP2 monoclonal antibody at 4°C for 12 h, followed by six washes and examined by Western blot assay. For the immunoprecipitation of VP2 or truncated EGFP-VP2(Δ1–Δ5) with endogenous CK2α, MDCC-MSB1 cells were transfected with pEGFP-N1, pEGFP-N1-*vp2,* or pEGFP-N1-*vp2*(Δ1–Δ5). Twenty-four hours post-transfection, cell lysates were harvested and subjected to immunoprecipitation with anti-GFP agarose beads at room temperature for 6 h, followed by six washes and examined with a Western blot assay. Based on identified essential regions, point mutations substituting residues in this domain with alanine were introduced into pEGFP-N1-*vp2* using the Q5 Site-Directed Mutagenesis Kit (NEB). All mutant constructs were verified by Sanger sequencing. Protein interaction assays were performed as described above.

### Confocal laser scanning microscopy assays

To determine the colocalization of VP2 and CK2α, MDCC-MSB1 cells were mock-infected or infected with CIAV at an MOI of 1. Twenty-four hours after infection, MDCC-MSB1 cells were fixed with 4% paraformaldehyde, permeabilized with 0.1% Triton X-100 for 15 min, and blocked with 1% bovine serum albumin in PBS for 1 h. Cells were then incubated with primary antibodies—mouse anti-VP2 and rabbit anti-CK2α—diluted in blocking buffer, overnight at 4°C. After washing, cells were incubated with secondary antibodies—FITC-conjugated goat anti-mouse IgG and TRITC-conjugated goat anti-rabbit IgG—for 30 min at room temperature, protected from light. After three washes with PBS, the nuclei were counterstained with DAPI for 5 min. Following three washes with PBS, coverslips were mounted onto glass slides. Images were acquired using a Leica TCS SP8 confocal laser scanning microscope (Leica Microsystems, Wetzlar, Germany).

### RNA isolation and reverse transcription-qPCR

Total RNAs were prepared from MDCC-MSB1 cells or tissues using an Rnafast200 Total RNA Rapid Extraction Kit (Fastagen, China), and then transcribed into cDNA by the HiScript III All-in-one RT SuperMix Perfect for qPCR (Vazyme, China) according to the manufacturer’s instructions. To quantify the mRNA expression of CIAV VP1, VP2, VP3, and cellular CK2α, qPCR was performed using 2× M5 HiPer SYBR premix EsTaq (Mei5 Biotechnology) on a LightCycler 480 II (Roche, USA). All samples were run in triplicate on the same plate, and the mean values were used for quantification. Primers used for qPCR were as follows: for CIAV VP1, sense primer 5′-CGACATCGGAGGAGACAGCG-3′ and antisense primer 5′- CCTTGGAAGCGGATAGTCATAGTAGA-3′; for CIAV VP2, sense primer 5′-ACGCTCTCCAAGAAGATACTCCACCC-3′ and antisense primer 5′-TTTAGCTCGCTTACCCTGTACTCGGAGG-3′; for CIAV VP3, sense primer 5′-CCCTCGAAGAAGCGATCCTG-3′ and antisense primer 5′-GGTCGGCTGGGAGTAGTGGTAA-3′; for CK2α, sense primer 5′-ATTGTGAAAGACCAGGCTCGGATG-3′ and antisense primer 5′-GGTGAAGGTGTTGGCACTGAAGAG-3′. All primers were designed and synthesized by Sangon Biotech Co., Ltd. (Shanghai, China). Thermal cycling parameters were as follows: 95°C for 30 s; 40 cycles of 95°C for 5 s and 60°C for 30 s; one cycle of 95°C for 5 s, 65°C for 1 min, and 95°C for 30 s. All samples were performed in triplicate on the same plate. Relative mRNA expression levels were calculated using the comparative cycle threshold (Ct) (2^−ΔΔCt^) method, normalized to the endogenous reference gene GAPDH, and expressed as fold change relative to the control group.

### Cell viability assay

MDCC-MSB1 cells were seeded on 96-well cell culture plates and cultured overnight. Cells were treated with different concentrations of the CK2α inhibitor CX-4945 (0.1, 1, 10, and 100 μM) or an equivalent volume of DMSO control. Twenty-four hours after treatment, 10 μL of CCK-8 reagent (Beyotime Biotechnology, Shanghai, China) was added to each well, followed by incubation at 37°C for 1 h, and the optical density of the solution was measured at 450 nm using a microplate absorbance spectrophotometer (Bio-Rad, USA). The results are representative of three independent experiments.

### DNA extraction and quantification of CIAV

To determine the effect of CK2α knockdown on CIAV infection, MDCC-MSB1 cells were transfected with 100 nM CK2α-targeting siRNA or negative control siRNA. Twenty-four hours after transfection, the cells were infected with CIAV at an MOI of 1. DNA was extracted at different time points (12, 24, 48, and 72 h) using a commercial DNA extraction kit. To determine the effect of CK2α inhibition on CIAV infection, MDCC-MSB1 cells were treated with 10 µM CX-4945 or an equal volume of DMSO as a control. Two hours after treatment, the cells were infected with CIAV at an MOI of 1. DNA was extracted at different time points (12, 24, 48, and 72 h) after infection. To determine the effect of the CK2α catalytic site on CIAV infection, CK2α expression levels were knocked down as aforementioned. After that, the cells were transfected with pEGFP-N1-CK2α, pEGFP-N1-CK2αK68M (a kinase-inactive mutant of CK2α), or pEGFP-N1 control plasmid. Twenty-four hours after transfection, the cells were infected with CIAV at an MOI of 1. DNA was extracted at different time points (12, 24, 48, and 72 h) after infection. To determine the effect of VP2 mutations on CIAV replication kinetics, MDCC-MSB1 cells were infected with CIAV-WT, CIAV-VP2/mutant, or CIAV-Revertant at an MOI of 1. DNA was extracted at different time points (12, 24, 48, and 72 h) after infection.

CIAV copy numbers were quantified by absolute fluorescent qPCR targeting the VP1 gene. Briefly, DNA concentration was adjusted to 100 ng/μL, and 1 μL (100 ng) was used per qPCR reaction with 2× M5 HiPer SYBR Premix EsTaq. Reactions were performed in triplicate on a LightCycler 480 II (Roche, USA). A standard curve was generated using serial ten-fold dilutions (10⁸ to 10¹ copies/mL) of a plasmid containing the CIAV VP1 gene fragment. CIAV genome copy numbers in samples were calculated based on the standard curve using the Ct values.

### Protein degradation assay

To investigate the effect of CK2α on VP2 stability, MDCC-MSB1 cells were transfected with CK2α-targeting siRNA or control siRNA and co-transfected with pRK5-flag-*vp2*. Twelve hours after transfection, protein synthesis was inhibited by adding CHX (50 μg/mL). Cells were harvested at different time points (0, 6, 12, and 24 h) and examined with Western blot using anti-FLAG, anti-CK2α, or anti-Tubulin antibodies. The levels of VP2 expression were quantified by densitometry and normalized to that of Tubulin. To investigate the effect of CK2 kinase activity on VP2 stability, cells were pretreated with 10 µM CX-4945 or DMSO control for 2 h and then transfected with pRK5-flag-*vp2*. Twelve hours post-transfection, CHX (50 µg/mL) was added. Lysates were collected at 0, 6, 12, and 24 h post-CHX and analyzed as above. To investigate the cellular pathway VP2 degradation, at 12 h post-transfection with pRK5-flag-*vp2*, cells were treated with CHX (50 μg/mL) alone, or in combination with the proteasome inhibitor MG132 (20 μM) or the lysosome inhibitor CQ (50 μM). Cells were harvested 12 h after treatment. Lysates were analyzed by Western blot (anti-FLAG, anti-Tubulin). To investigate VP2 stability during viral infection, MDCC-MSB1 cells were infected with CIAV-WT, CIAV-VP2/mutant, or CIAV-Revertant (MOI = 1). At 24 h post-infection, CHX (50 μg/mL) was added. Cells were harvested at 0, 6, 12, and 24 h post-CHX treatment. Lysates were analyzed by Western blot (anti-VP2, anti-Tubulin). VP2 levels were quantified and normalized as described. All experiments were performed in triplicate.

### Construction of CIAV infectious clones

The complete genome of CIAV-WT (Cux-1 strain) was extracted. The viral DNA was subsequently utilized to amplify the CIAV genome using Q5 High-Fidelity DNA Polymerase (NEB). The CIAV genome was cloned into the pcDNA3.1 vector via homologous recombination. The recombinant plasmid, pcDNA3.1-CIAV, was created by inserting an AflII polyclonal site as a genetic marker at each end of the genome. The pcDNA3.1-CIAV plasmid served as a template for the mutation of VP2, generating the mutant plasmid pcDNA3.1-CIAV-VP2/mutant. This mutant plasmid was then used as a template to produce the restored mutation plasmid pcDNA3.1-CIAV-Revertant. All plasmids were verified by Sanger sequencing. Primer sequences used for plasmid construction are available upon request.

### Rescue of CIAV mutant viruses

The CIAV genomic insert was released from the pcDNA3.1-CIAV plasmid by digestion with AflII restriction enzyme. The linear DNA fragment was purified using the High Pure PCR Product Purification Kit (Magen, Guangzhou, China). The purified genomic DNA was self-ligated using T4 DNA Ligase (NEB) to generate circular viral genomes. MDCC-MSB1 cells were transfected with the ligated DNA using the electroporation method as previously described. Transfected cells were cultured for 72 h. Cells were then subjected to three freeze-thaw cycles at −80°C in order to harvest the rescued virus. The virus was then blindly passaged in MDCC-MSB1 cells for three generations before characterization. Similarly, the infectious clones pcDNA3.1-CIAV-VP2/mutant and pcDNA3.1-CIAV-Revertant were used to generate the rescue viruses CIAV-VP2/mutant and CIAV-Revertant, respectively.

### Statistical analysis

Statistical analyses were performed using GraphPad Prism version 7.0. Data are presented as the mean ± SD from at least three independent experiments, each performed with three replicates. Differences between two groups were assessed using the unpaired two-tailed Student’s *t*-test. Comparisons among multiple groups were analyzed by one-way analysis of variance followed by Tukey’s *post hoc* test. A *P*-value less than 0.05 was considered statistically significant. In the figures, statistical significance is indicated as follows: ***, *P* < 0.001; **, *P* < 0.01; *, *P* < 0.05.

## Data Availability

The data that support the findings of this study are available from the corresponding author, upon reasonable request.

## SUPPLEMENTAL MATERIAL

Fig. S1 jvi.01739-25-s0001.doc CK2α overexpression does not affect CIAV VP2 expression or viral replication.

Table S1 jvi.01739-25-s0002.xls Proteins identified by LC-MS/MS analysis of VP2 immunoprecipitates.

Table S2 jvi.01739-25-s0003.xls Peptides identified by LC-MS/MS analysis of VP2 immunoprecipitates.

ASM does not own the copyrights to Supplemental Material that may be linked to, or accessed through, an article. The authors have granted ASM a non-exclusive, world-wide license to publish the Supplemental Material files. Please contact the corresponding author directly for reuse.
