# Supplementary material for: Chicken infectious anemia virus exploits host CK2α as an essential factor for its replication via VP2 Ser182/Asp183-mediated interaction
Source: J Virol. 2026 Mar 31;100(4):e01739-25. doi: 10.1128/jvi.01739-25 (PMC13098270; doi:10.1128/jvi.01739-25)
Supplement: Fig. S1 — CK2α overexpression does not affect CIAV VP2 expression or viral replication. [file jvi.01739-25-s0001.doc]

**Supplemental figure**


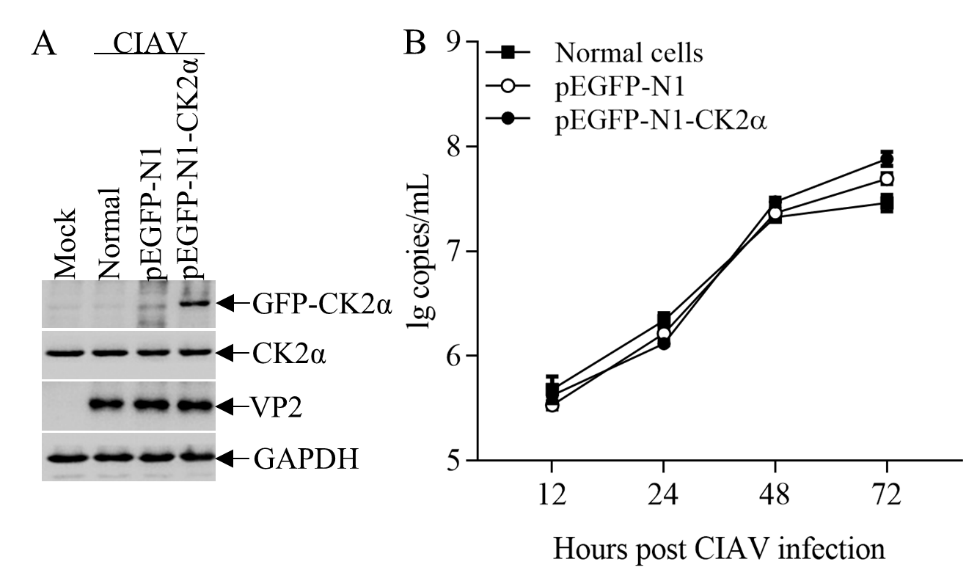


**Figure S1.** **CK2α overexpression does not affect CIAV VP2 expression or viral replication.** (A) Effect of CK2α overexpression on CIAV VP2 expression. MDCC-MSB1 cells were transfected with pEGFP-N1-CK2α or pEGFP-N1, then infected with CIAV (MOI = 1). (B) Impact of CK2α overexpression on CIAV growth kinetics. MDCC-MSB1 cells were transfected with pEGFP-N1-CK2α or pEGFP-N1, then infected with CIAV (MOI = 1). Viral genome copies were quantified by qPCR at 12, 24, 48, and 72 hpi.
